# Iodide of Lead Ointment

**Published:** 1871-02

**Authors:** 


IODIDE OF LEAD OINTMENT.—Dr. Purdon thinks this ointment is not fully appreciated by dermatologists. He esteems it very useful in some varieties of psoriasis, in tinea circinata, and in scrofulous affections.

